# A mixed-methods investigation of women’s experiences seeking pregnancy-related online nutrition information

**DOI:** 10.1186/s12884-020-03065-w

**Published:** 2020-06-26

**Authors:** Alexandra Snyder, Hannah Tait Neufeld, Laura Forbes

**Affiliations:** 1 Department of Family Relations and Applied Nutrition, University of Guelph, 50 Stone Rd., Guelph, ON N1G 2W1 Canada; 2 School of Public Health and Health Systems, University of Waterloo, 200 University Ave W, Waterloo, ON N2L 3G1 Canada

**Keywords:** Pregnancy, Internet, Health education, Diet, food and nutrition

## Abstract

**Background:**

The objective of this study was to describe women’s processes for finding pregnancy-related nutrition information, their experiences seeking this information online and their ideas for improving internet sources of this information.

**Methods:**

In total, 97 pregnant women completed an online quantitative questionnaire and 10 primiparous pregnant women completed semi-structured telephone interviews. Questionnaires and interviews asked participants to describe sources of pregnancy-related nutrition information; time of seeking; processes of searching online; experiences searching online; ideas for improving information found online. Survey data were analyzed using descriptive statistics and Chi square tests; interview data were analyzed using thematic analysis.

**Results:**

Nearly all (96%) survey participants sought nutrition information online. Information was most commonly sought during the first trimester of pregnancy. Motivators for using the internet included convenience and lack of support from health care providers. Barriers to using online information included lack of trust, difficulty finding information and worry. Women adapted the information they found online to meet their needs and reported making positive changes to their diets.

**Conclusions:**

The internet is a key source of prenatal nutrition information that women report using to make positive dietary changes. Women would benefit from improved access to trustworthy internet sources, increased availability of information on different diets and health conditions, and increased support from health care providers.

## Background

Suboptimal dietary intake during pregnancy is associated with a variety of negative health outcomes for both the mother and her baby, including gestational diabetes mellitus, excessive or inadequate gestational weight gain, preterm birth, and poor cognitive development in the baby [1–4]. A diet containing foods with an appropriate macro- and micronutrient content, however, can reduce the risk of adverse outcomes [1–6].

Studies of dietary intake of pregnant women in Canada show that women frequently fail to meet food group recommendations from *Eating Well with Canada’s Food Guide* and that, on average, diet quality is low [7–9]. Moreover, 49% of Canadian women gain excessive weight during pregnancy in relation to current guidelines [10], suggesting that excessive caloric intake is common during pregnancy for this population group [11].

Research on prenatal nutrition care in Canada has shown that women receive little education from their health care providers on consuming a healthy diet during pregnancy or on appropriate gestational weight gain [12, 13]. As a result, many women may turn to other sources for this information such as the internet, books, pamphlets, friends and family [14, 15]. The information seeking behaviours of Canadian pregnant women, however, have not been researched and may be unique. Studies in other higher resource countries have found the internet to be the primary source that women use for pregnancy information [14–18]. Therefore an in-depth investigation of women’s experiences with internet sources of information is warranted.

The objectives of this study were to 1) describe women’s processes for finding pregnancy-related nutrition information; 2) describe their experiences using the internet to find this information and; 3) explore how these sources could be improved.

## Methods

A mixed methods approach was used to explore the experiences of women seeking prenatal nutrition information. A quantitative web-based survey in conjunction with qualitative semi-structured telephone interviews were used to achieve the first objective, and the interviews alone were used to achieve the last two objectives. Prior to the start of the study, ethical approval was obtained from the University of Guelph Research Ethics Board (REB) and all participants provided informed consent before participating in the study. Consent was completed electronically by survey participants and verbally by phone interviewees as approved by the REB.

### Participants

Women were eligible to participant in the study if they were 18 years of age or older, resided in Ontario, Canada, and were able to read and speak English. The women who participated in the survey had to be pregnant at the time of completion. Interview participants were allowed to be pregnant or up to 3 months post-partum, as some were recruited from the online survey and had given birth by the time of their interview. Only primiparous women were eligible to participate in the interviews, as previous research has shown that women seek more information during their first pregnancy than in subsequent pregnancies [19].

### Recruitment

Ninety-seven participants were recruited for the survey via posters in community health centres, public health centres, Canada Prenatal Nutrition Programs and midwifery practices, and via online posts on social media sites and online pregnancy forums. A goal of approximately 100 participants was set for the survey based on similar sample sizes in previous studies investigating pregnant women’s preferred information sources [19–21]. Six women were recruited for interviews via the online survey, and four additional women were recruited using snowball sampling. Recruitment of interview participants continued until data saturation was reached. Women were not compensated for their participation.

### Instrument development

The survey and interview guide were developed collaboratively by the research team. A member of the research team (HTN) with experience in survey and interview guide development reviewed the survey and interview questions to ensure they had face validity and aligned with the research questions. The survey was piloted tested with two pregnant women and the interview guide was pilot tested with one pregnant woman prior to the start of the study. One issue was noted with a link in the survey, which was fixed, and no changes were made to the interview guide as only positive feedback was provided.

### Data collection procedures

The quantitative survey was administered using the online survey software, Qualtrics, and data collection occurred over a 7 month period [22]. The survey collected demographic information, including age, gestational age, parity, ethnicity, marital status, education and income (See supplementary material for a copy of the survey). The survey then asked women to indicate all the sources they used to find prenatal nutrition information, which source they used most often, when they looked for information and when they looked most often. Women in their first pregnancy were asked if they would like to take part in a telephone interview at a later date.

All interviews were conducted by one researcher (AS). In the telephone interview, participants were asked to describe how they looked for prenatal nutrition information, including where they looked (i.e. search engines, specific websites) and what nutrition topics they looked for online. They were then asked to describe their experiences searching for nutrition information online including their reasons for using the internet, their success with finding information, their understanding of the information, emotional responses and whether the information they found resulted in changes to their thinking or behaviour. Finally, the women were asked for their ideas about how internet-based nutrition information could be improved (See supplementary material for a copy of the interview guide).

### Data analysis

The statistical software package SPSS (version 22.0, SPSS Inc., Chicago, IL, 2016) was used for statistical analyses. Descriptive statistics were used to determine which sources of prenatal nutrition information were used most often and when women were looking for information. Chi-square tests were used to determine if participants’ age, ethnicity, marital status, education, income, stage of pregnancy and parity influenced the sources consulted or time of seeking nutrition information. *P*-values ≤0.05 were considered statistically significant.

The interviews were audiotaped and transcribed verbatim. The participants were given pseudonyms to ensure anonymity. Data were analysed using NVIVO software (version 11, QSR International Pty Ltd., 2015). Thematic analysis was used to analyze the data [23–26]. Initial codes were generated by one researcher (AS). From these codes, themes and a thematic map were created showing relationships between themes [25]. The map was reviewed by the whole study team and revised until all the data fit well into the thematic map.

## Results

### Participants

Ninety-seven women completed the online survey. Demographic data (Table 1) show that participants primarily identified as European, Canadian or White (94%), were married (96%) and were between the ages of 26 and 35 (74%). Most women had post-secondary education (97%) and had incomes over $80,000/year (68%). In total, 41% were primiparous, and while all three trimesters of pregnancy were represented, 45% of women were in their third trimester. Our sample contained a higher proportion of women of European descent and women with post secondary education when compared to all women in Ontario [27, 28], and participants had higher incomes than the average family in Ontario [29]. The chi-square tests revealed no significant relationships between demographics and the sources women consulted or when they looked for nutrition information.

**Table 1:** Demographic data of participants of a study examining nutrition information-seeking behaviours during pregnancy

Characteristic	Number of Survey Participants (%) (***n*** = 97)	Number of Interview Participants (%) (***n*** = 10)
Pregnancy number		
First pregnancy	40 (41)	10 (100)
Have been pregnant before	57 (59)	0 (0)
Stage of pregnancy		
0–3 months	28 (29)	0 (0)
3–6 months	25 (26)	4 (40)
6–9+ months	43 (45)	2 (20)
< 3 months postpartum	0 (0)	4 (40)
Age		
18–25	11 (12)	1 (10)
26–35	70 (74)	9 (90)
36–45	14 (15)	0 (0)
Ethnic background		
First Nations/Métis/Inuit	7 (7)	0 (0)
European/Canadian/White	88 (94)	10 (100)
Other	5 (5)	0 (0)
Marital status		
Legally married/Common-law married	91 (96)	9 (90)
Never legally married/divorced/widowed	4 (4)	1 (10)
Education		
Completed high school	3 (3)	0 (0)
Post secondary education	67 (71)	5 (50)
Graduate education	25 (26)	5 (50)
Annual household income		
0-$39,000	8 (9)	2 (20)
$40,000–$59,000	10 (11)	0 (0)
$60,000–$79,000	12 (13)	0 (0)
$80,000–$100,000	26 (28)	3 (30)
$100,000+	37 (40)	4 (40)
Prefer not to answer	0 (0)	1 (10)

The participants were asked to choose all that apply for their ethnic background

A total of 10 interviews were conducted, which were between 12 and 20 min in length. Data saturation was reached in the last two interviews. Demographic characteristics of interview participants are shown in Table 1. The participants ranged in age from 23 to 35, identified as Canadian or European, and lived in a variety of urban and rural locations across Ontario. All participants had post-secondary education and the majority were married with a household income over $90,000/year. Six of the participants were still pregnant at the time of their interview, while the other four were less than 3 months post-partum.

### Processes of searching for pregnancy-related nutrition information

**Fig. 1:**
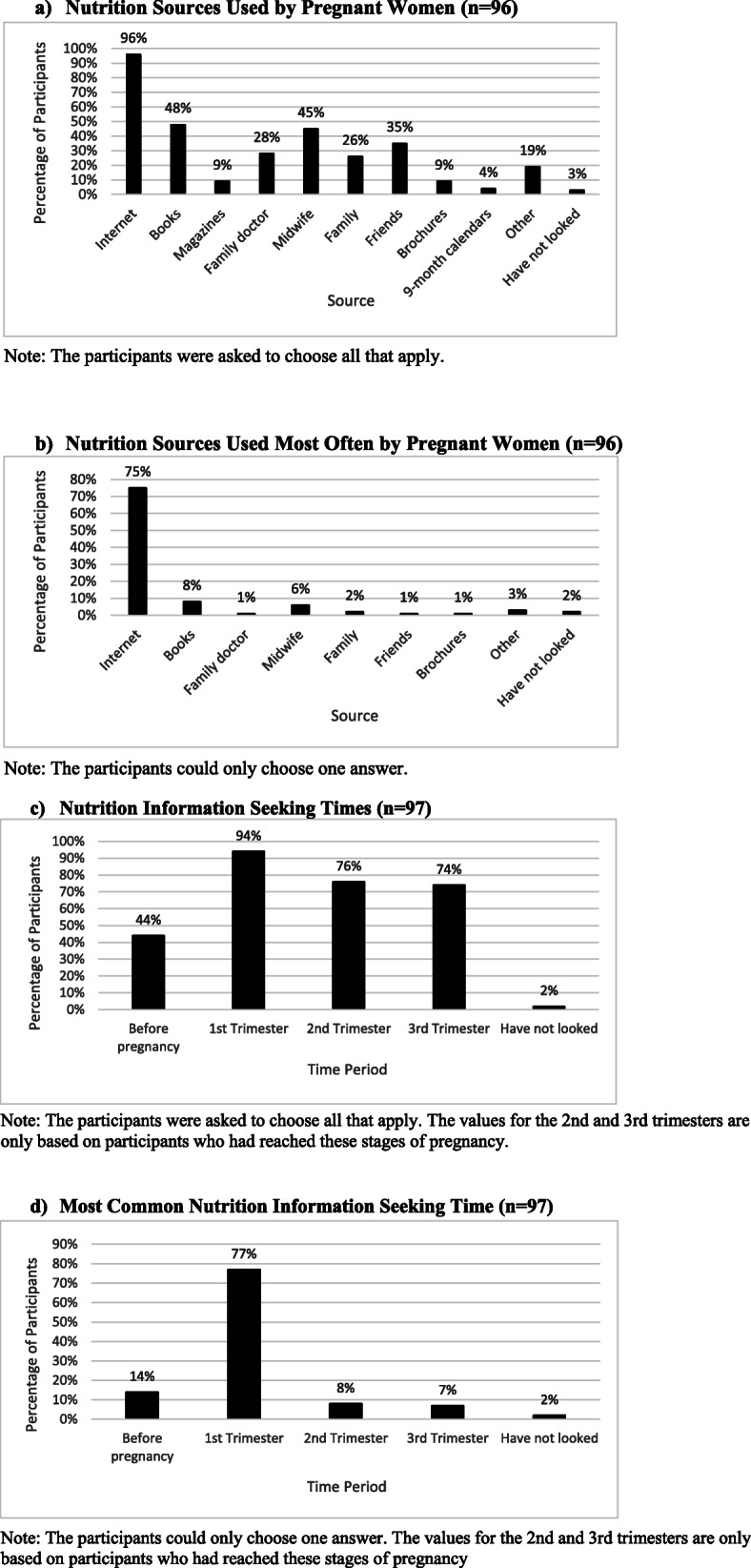
Sources and Times that Pregnant Women Sought Nutrition Information

In total, 96% of survey participants used the internet to find pregnancy-related nutrition information (Fig. 1a) and 75% reported they used the internet more often than any other source (Fig. 1b). Similarly, the internet was the most popular source of information among the interview participants.

The interviewees reported using the internet to research a variety of nutrition topics relating to pregnancy. Topics that women discussed searching for online included: iron, protein, calcium and dairy, caffeine, energy requirements, serving sizes, recipes/meal plans, and foods to avoid.

When interview participants were asked to describe their process of searching for nutrition information online, they all reported using Google searches. Some women would visit the first few websites that came up in the search results, while others would choose websites from the list that they perceived to be credible. Claudia described her search process: “*I’ll just Google…goat cheese and pregnancy and then I usually pick from the top results but I try to look at what kind of a website it is*.” Common websites that participants reported visiting included: Baby Centre, Pinterest, Government of Canada websites (including Canada’s Food Guide), The Bump, What to Expect When You’re Expecting, websites by Dietetic Associations, the Canadian Cardiovascular Society and Web MD.

Most survey participants (74%) looked for nutrition information in all trimesters of pregnancy (Fig. 1c), and 77% looked most often during their first trimester (Fig. 1d). Likewise, when asked about when they used the internet to find pregnancy-related nutrition information, the interview participants described seeking this information most frequently in early pregnancy, but they continued to look throughout their pregnancies as questions or problems emerged (i.e. developed heartburn, had excess weight gain). Less than half of the survey participants looked for nutrition information prior to becoming pregnant. This was reflected in the interviews as well, as only two interviewees discussed seeking prenatal nutrition information before pregnancy.

### Women’s experiences seeking pregnancy-related nutrition information online

**Fig. 2:**
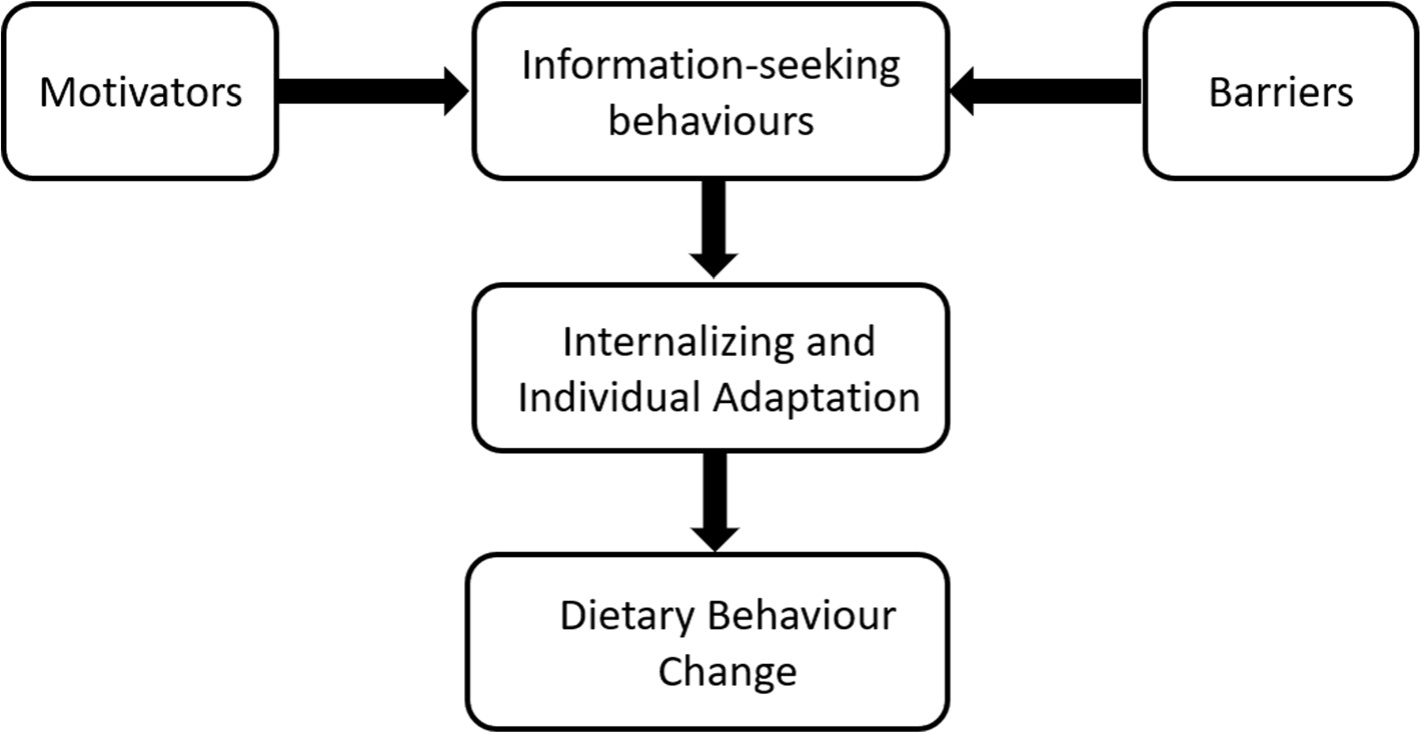
Thematic Map Describing Women’s Experiences in Searching for Online Nutrition Information During Pregnancy

When women were asked in the interviews to describe their experiences finding pregnancy related nutrition information on the internet, three overarching themes emerged as shown in Fig. 2. The main themes were: Motivators for using the internet; Barriers to using the internet; and Internalizing and individual adaptation. These themes are described below.

#### Motivators for using the internet

During the interviews, the participants were asked about their reasons for using the internet, and convenience and lack of support from health care providers were described as key motivators. All of the women mentioned convenience as an important motivator because, unlike other sources of nutrition information, the internet provided immediate answers at any time or location. Jodie said: *“I could do it when I needed to so when I was still at work and other things like that.”* Maryam provided a specific example illustrating the convenience of an instant answer: *“I had thought herbal teas were fine, only then to be out with my sister and a colleague of hers said, ‘Oh, actually that’s one you should avoid!’… and so, again, standing in line in the aisle at the grocery store googling quickly, ‘Oh, yes, is this good for pregnancy or not?’”*

While a few women reported receiving helpful nutrition advice from their health care providers, several reported receiving insufficient nutrition information, which led them to use the internet to fill this gap. For example, Ariella said: *“I was imagining you’d get pregnant and your doctor would give you a whole bunch of pamphlets on like how to eat and which foods to avoid but none of that really happens, you’re kind of left to your own devices, and so I did searching a little bit online at the beginning.”* Another participant reported asking for dietary support but did not find her midwife’s answers helpful: *“I would email my midwife… if I had any questions but a lot of the times their answers were more either generic based on the answers from Health Canada…or just kind of saying that it’s a personal choice or a personal decision”* (Nadine). In addition, some women felt that their health care providers were not able to provide advice as quickly as desired, therefore the internet was a preferred source.

#### Barriers to effective use of the internet

When discussing their experiences using the internet, women were asked if they were able to find the information they were looking for, and if it aligned with information they received from other sources. They were also asked whether their online searches brought up any emotions for them. Through these questions, three key barriers to effective use of the internet emerged, including the need to double-check internet sources for trustworthiness and accuracy, difficulty finding desired information, and feelings of worry associated with information found online. Women perceived many internet sources of information to be untrustworthy. For example, Amanda said: *“It wouldn’t come up with websites right away that were valid websites…a lot of it is forums or group discussions…so I don’t really know if that’s valid because that’s literally just someone random wrote it.”* Women also identified websites with contradictory information as being untrustworthy. To address issues of trustworthiness, women would look for answers in more than one place before deciding what to believe. Some women read multiple websites on the same topic. For example, Claudia said: *“I know I can find answers that are saying something is ok and something is terrible for you at the same time so that’s why I try to look at maybe two or three different sites.”* Other women consulted an additional source, such as books or their care provider, after searching the internet. For example, Jodie said: *“If I found contradictory information then I would probably refer to either my doctor or my family member who is a doctor to try and confirm the contradictory information and find out what was actually accurate.”*

Some women had difficulty finding information on the internet that they wanted, including Canadian information, information about specific health conditions in pregnancy, and information about specific dietary needs. For example, a participant who followed a vegetarian diet had trouble finding suggestions for increasing plant-based protein intake. She reported *“digging through a lot of information that didn’t apply to [her] and [her] diet”* (Rebecca). In addition, some women found it challenging to find a single website with all of the information that they needed. Amanda said: *“It never was the first website that would pop up that would be the perfect website.”* Some women also had difficulty accessing information from certain websites on their phones. *“[T]he medical websites, they’re not as mobile friendly, some of them would just not load correctly or you have to refresh the page.”* (Dana).

A few participants mentioned feeling worried about their dietary habits in relation to information that they found online. Nearly everyone looked up foods to avoid during pregnancy, and women expressed concern after finding out they had eaten something bad for their baby. Some participants worried that they would not be able to provide their growing babies with enough nutrients. For example, Rebecca became concerned when she could not find much information online about getting enough protein with a vegetarian diet: *“You see so many things recommending all of these meat sources [and] sometimes it kind of can get you to question yourself, or make you concerned that you’re not going to meet your weight gain targets.”*

#### Internalizing and individual adaptation

Within the theme of internalizing and individual adaptation, the sub-themes of self-confidence and positive change described how women took the information they found online and adapted it to make decisions and meet their unique dietary needs during pregnancy. Self-confidence appeared to be a quality that helped women effectively use the internet to make dietary decisions. This emerged as a theme through a variety of questions asked, including how women found information online, if the information was clear and easy to understand, and if information they found online changed their previous beliefs about nutrition during pregnancy. Several participants said they would do their research but ultimately trusted themselves to decide what to eat during pregnancy. Jodie explained that she searched the internet, then *“made the personal position on what I thought I was going to do and did that.”* Some participants explained that they were confident due to previous education. For instance, Francesca said: *“I have a nursing background so I…kind of know a little bit about what I’m supposed to be eating.”* Others discussed gaining confidence throughout pregnancy. Amanda explained how at first, she tried to adhere to all the healthy eating recommendations for pregnancy but *“learned throughout the nine months that it wasn’t the end of the world if you didn’t follow your perfect nutrition that it says online or it says in a book.”*

During the interviews, women were asked if anything they came across on the internet changed the way they ate during pregnancy. All of the women interviewed reported making changes to their diets using information they found online. Types of changes that women reported making included avoiding unsafe foods, increasing their intake of individual nutrients and changing their diets to address specific health conditions or pregnancy symptoms. Some food items participants said they avoided were certain types of fish, herbal teas, uncooked egg yolk, soft cheeses, deli meats, hotdogs, alcohol, and caffeine. Nadine explained how she increased her nutrient intake: *“I had anemia so I would use* [the internet] *to find out foods that had higher iron content and then find different recipes that could allow me to ingest more iron.”* Dana explained how she used the internet to manage her pregnancy symptoms: *“I always thought especially for heartburn having dairy like yogurt or milk or something would actually help soothe it but it was definitely something that I found on the internet saying stay away from that because it can actually make it worse.”*

### Ideas for improvement

The final interview question asked women what changes they would make to the prenatal nutrition information available online. Two main recommendations for improvement were made. The first suggestion was for more information to be available for women with different lifestyles or health conditions. Amanda said: *“I think it needs to be broken down a lot more…like if you’re this lifestyle or if you’re this size or if you have these problems you should be eating more like this.”* The second suggestion was for simplified access to credible information online: *“*[It] *would have been easier if there was somewhere where it was recommended moms go here because this is where your most up-to-date information is going to come from”* (Lucia). Maryam agreed that it was hard to find credible internet sources but believed it would be more practical to encourage health care providers to *“make sure that clients have good digital literacy skills”,* rather than create a new space online with credible information.

## Discussion

This study has identified that the internet is a key source of prenatal nutrition information for pregnant women in Canada and that women report changing their eating habits as a result of information they find online. However, women reported that credible information is not always easy to find or comprehensive enough to meet their needs. These findings suggest that there is an opportunity to create better resources of online prenatal nutrition information, as well as improve the support provided by health care professionals.

The results of this study show that pregnant women in Canada have similar information finding techniques and experiences as women in other countries and people searching for nutrition or medical advice in other life stages [14–17, 19, 30]. Similar to other studies [15, 16, 19], women in our study used the internet because it was fast and convenient and because they received insufficient advice from their health care practitioners. Key barriers to using online sources, including the trustworthiness of sources and the worry experienced by women when they find answers to their questions has also been described previously in other populations [31–34]. While these findings are not new, they emphasise that interventions aiming to target this population will need to ensure a nutrition in pregnancy website is easy to find using common search engines, is trustworthy and up-to-date and that women experiencing worry will have access to supports such as a health care practitioner to discuss any concerns.

Another important finding of this study was that some women had difficulty finding information for their specific dietary needs. Women who followed certain dietary patterns, such as vegetarianism, or who had health conditions that impacted their nutritional requirements struggled to find adequate advice online. A recent study in Australia also found that some pregnant women had trouble finding information specific to their personal needs [35]. This suggests that an ideal online pregnancy nutrition resource would contain information for women with a wide variety of nutrition needs or concerns. Discussion with a health care professional may also prove more useful in some situations as they have the potential to provide more individualized care.

Some women in the current study shared their desire for better access to credible information online, and suggested that health care providers assist with this. Similarly, Huberty et al. [14] found that women wanted their health care providers to assist them with finding pregnancy information online. The suggestions from women in the current study could be used to guide the creation of prenatal nutrition websites or health care interventions that would improve women’s access to credible nutrition information. Health care providers could also play a greater role in guiding women in their online searches.

Our study also identified some new findings, including information about the types of behaviours women appeared to change based on online sources, and the importance of self confidence in helping women make decisions based on information they read online. While previous research has found that online pregnancy information aids women in making decisions and changing their lifestyles [14, 16, 34], this study is the first to find that the internet specifically helped women make perceived changes to their diets, including increasing their intake of certain nutrients and plant-based foods, avoiding unsafe foods and maintaining a healthy diet throughout pregnancy. These new findings may be indicative of modern nutrition trends both in terms of the types of information available from online sources and the types of information women are searching for.

This study identified self-confidence as a key trait women needed in order to make decisions and perceived changes to their dietary intake during pregnancy in relation to information they read online. Some women in the current study were confident in their ability to make nutrition-related decisions during pregnancy, and others gained confidence throughout their pregnancies. A positive relationship has been shown between self-efficacy and the use of online health information in previous studies [36, 37]. Thus, interventions that build women’s self-confidence could aid them in using internet-based information to make positive changes to their diets.

### Strengths and limitations

A key strength of this study was the mixed-method approach, which collected numerical data from a larger number of women, and contextualized the quantitative findings using interview data. Another strength was that the interviews reached saturation with no new themes emerging in the last two interviews. One limitation of the study was that there was a lack of diversity in the sample population, which consisted of primarily white, married women with high socioeconomic status (SES). The findings are only generalizable to women of these demographics; however it is logical to assume that the challenges identified in this study may be magnified in disadvantaged populations. For example, women with lower SES may have more difficulty finding credible online sources, and they may require more support from their health care providers. Future research should focus on women of low SES to determine how their experiences seeking prenatal nutrition information differs and how it can be improved. Another limitation of the study was that the use of an online questionnaire may have led to a response bias, favouring women who are frequent internet users. In addition, some participants were pregnant during data collection and were not able to reflect on their entire pregnancies, however this reduced the likelihood of recall bias.

## Conclusions

This study showed that the internet was an important source of nutrition information during pregnancy, and women used the information found online to make positive dietary changes. The internet was of particular importance as a source of nutrition information early in pregnancy, but continued to be used throughout. Some key barriers to effectively using internet sources of nutrition information were identified, including trustworthiness of information, challenges finding specific information and worry associated with searching the internet. To combat these barriers, four main areas for improving women’s nutrition information-seeking experiences were identified:

1. Improve the availability of tailored information for different groups of women from internet sources.
2. Improve access to trustworthy sources of internet information.
3. Increase nutrition support from health care providers, including assistance with finding and interpreting information from internet sources.
4. Develop interventions to increase women’s self-confidence in finding trustworthy information.

Interventions or programs that target these recommendations could improve women’s experiences seeking nutrition information and support them in making positive behaviour changes during pregnancy.

## Supplementary information

**Additional file 1: Supplementary Material 1.** Interview Guide and Consent Script. Introductory phone script that includes study information along with demographic and interview questions as part of the qualitative component of the research.

**Additional file 2: Supplementary Material 2.** Study information and consent details included in addition to all close-ended survey questions that made up the online questionnaire for the research.

## Data Availability

The datasets generated and/or analysed during the current study are not publicly available as permission to grant public availability of the dataset was not given in the original consent forms for this study.
