# Supplementary material for: A mixed-methods investigation of women’s experiences seeking pregnancy-related online nutrition information
Source: BMC Pregnancy Childbirth. 2020 Jun 26;20:377. doi: 10.1186/s12884-020-03065-w (PMC7320538; doi:10.1186/s12884-020-03065-w)
Supplement: Supplementary file 1 — Additional file 1: Supplementary Material 1. Interview Guide and Consent Script. Introductory phone script that includes study information along with demographic and interview questions as part of the qualitative component of the research. [file 12884_2020_3065_MOESM1_ESM.docx]

# Interview Guide and Consent Script

## Introduction & Study Information

Hello, may I please speak to [*name of participant*]? Hi, this is Alexandra Snyder calling from the University of Guelph. I’m calling for the interview we scheduled to talk briefly about your experiences with looking up pregnancy information online. Is this a good time for you?

(*Great* or *when would be a good time to call back?*).

Thank you for taking the time to do this, I really appreciate it. If you get another phone call or something comes up at any time during the interview just let me know and we can pause and finish at another time.

Did you receive an email outlining the purpose of the study and what to expect from the interview? I will take a few minutes to go over the main points again briefly:

- The purpose of the interviews is to gain a better understanding of women’s experiences using the internet to find pregnancy-related nutrition information.
- The interview is expected to last approximately 30 minutes to an hour, depending on the length of your answers.
- The interview will be audio recorded so the information can be reviewed later.
- Your participation in this interview is completely voluntarily. You can choose to withdraw at any time during the interview without any consequences, and you can skip any questions that you do not wish to answer.
- If you choose to withdraw from the interview, the recording of your responses will be destroyed upon request.
- There are no known or anticipated risks to participating in this study.
- During analysis, a pseudonym or another name of your choosing will be used in place of your real name. Any identifying information will be kept in a master list, separate from the data you provide; all information will be kept on an encrypted and password protected laptop, and all recordings of interviews will be deleted in the fall of 2017.
- Every effort will be made to ensure the confidentiality of identity of participants.

Do you have any questions before we begin?

(*Begin recording*). **Do you agree to participate**? Alright then we will start.

## Demographic Questions:

I’m going to begin by asking you a few questions about yourself.

***Question 1:*** Can you tell me what year you were born?

***Question 2:*** Can you tell me a little bit about your family’s background?

***Question 3:*** What area of Ontario do you currently live in? (Probes: Northern, Eastern, Southwestern, etc.)

***Question 4:*** Can you tell me the highest level of schooling/education that you have completed? (Probes: high school, college/university, graduate school, etc.)

***Question 5:*** Can you tell me what your approximate annual household income would be?

***Question 6:*** How would you describe your relationship status? (Probes: married, single, common-law, etc.)

Great thank you. Now we will move on to the pregnancy-related questions.

## Interview Questions

***Introduction Question:*** So how is the pregnancy going so far?

***Question 1:*** How far along are you in your pregnancy now?

***Question 2:*** Can you tell me about how you went about finding nutrition information during your pregnancy? (Probes: physician, midwife, family, friends, books, internet, brochures, etc.)

***Question 3:*** Have you used the internet to find pregnancy-related nutrition information? Can you give me some examples of when you used the internet to find this information? (Probes: ask about timing if they do not mention it – before pregnancy, 1^st^, 2^nd^, 3^rd^ trimester).

***Question 4:*** How/where did you find pregnancy-related nutrition information on the internet? (Probes: Search engine, hospital website, government website, chat room, etc. – ask for specific websites if they don’t say)

***Question 5:*** Why did you decide to use the internet to find this information? (Probes: Anonymity, speed, ease of access, cost, etc.)

***Question 6:*** How would you describe your experiences using the internet to find this information? (Probes: Were you able to find what you were looking for? Did it align with what you heard from other sources?)

***Question 7:*** Was the information you found on the Internet clear and easy to understand? Why? Did you find the websites user friendly? What made them easy or hard to use? (Probes: any big medical words, links to more information, easy to navigate).

***Question 8:*** Do you have any other family members or friends that are pregnant? Would you recommend the internet as a good source of nutrition information to them? (Probe: any specific websites or topics?)

***Question 9:*** Did you find that doing searches for nutrition-related information on the internet has brought up any emotions for you? If so, can you describe those experiences?

***Question 10:*** Did the information you found on the internet change your previous thoughts or beliefs about eating and nutrition during pregnancy? If yes, in what way?

***Question 11:*** Did anything you came across on the internet change the way you ate during pregnancy? If yes, can you give me a few examples?

***Question 12:*** If you could make changes to the nutrition information found on the internet that is available to pregnant women what would you do?

(Probes: Readability, pictures, examples, definitions of words, references to other sources, etc.)

***Closing questions:*** Is there anything we haven’t touched on today that you would like to tell me about? Do you have any questions for me?

***Debriefing:*** Thank you very much for taking the time to complete this interview. If you have any questions or concerns that come up later don’t hesitate to contact me, or if you are interested in receiving a summary of the research findings, please send me an email and I would be happy to provide you with that when the study is completed.
