# Supplementary material for: A mixed-methods investigation of women’s experiences seeking pregnancy-related online nutrition information
Source: BMC Pregnancy Childbirth. 2020 Jun 26;20:377. doi: 10.1186/s12884-020-03065-w (PMC7320538; doi:10.1186/s12884-020-03065-w)
Supplement: Supplementary file 2 — Additional file 2: Supplementary Material 2. Study information and consent details included in addition to all close-ended survey questions that made up the online questionnaire for the research. [file 12884_2020_3065_MOESM2_ESM.docx]

#
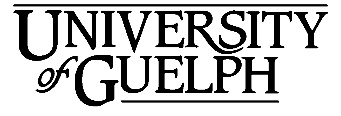
Online Questionnaire & Information Letter

##

## Section 1: Study Information & Consent

This online survey is the first part of a research study being conducted by a Masters of Science student at the University of Guelph. The purpose of this study is to better understand the experiences of pregnant women in Ontario with seeking nutrition information. Your responses to this survey will provide insight on what sources pregnant use to find nutrition information, and when they are searching for this information.

The survey should take approximately 10 minutes to complete. You will be asked 5 survey questions, followed by collection of some demographic information. There are no known or anticipated risks involved with participating in this survey. Your decision to participate or not participate will have no impact on your relationship with or services provided by the organization through which you heard about this study. Participation is voluntary and you may decide to withdraw at any point by closing your browser. You may also skip any questions that you do not wish to answer, without any consequences. You will only be re-contacted if you wish to take part in an interview at a later time or wish to receive a summary of the research findings, in which case you may leave your email address in the space provided on the survey. Your responses will only be sent to the researcher when you click “submit” at the end of the survey. After submission of the survey there will be no way to withdraw your answers because no identifying information will be linked to your responses.

Upon completion, you will receive a list of credible online nutrition information sources for pregnancy. This study will also benefit pregnancy research in Canada by providing information about the types of sources pregnant women in Ontario are using to find pregnancy-related nutrition information, and when they are looking for this information; this information is not currently available in Canada. In addition, it is hoped that the information obtained through this study will be used to improve the information available to pregnant women in Ontario.

This survey is anonymous, meaning at no time will any identifier be associated with the data you provide. Your email address and demographic information will be collected using a separate, unlinked survey to ensure the confidentiality of your identity. The research team will not release any information which identifies you when disseminating the final results. However, because data collection occurs via the internet, complete confidentiality of the data cannot be guaranteed. If you use public computers to fill out a survey, erase the history, empty the cache, and close the browser to help keep your information secure.

This project has been reviewed by the University of Guelph Research Ethics Board for compliance with federal guidelines for research involving human participants. If you have any questions regarding your rights and welfare as a research participant in this study (REB #16OC023), please contact: Director, Research Ethics; University of Guelph; reb@uoguelph.ca; 519-824-4120 ext. 56606. You do not waive any legal rights by agreeing to take part in this study. You may also contact: Alexandra Holt, student researcher, Department of Family Relations and Applied Nutrition, University of Guelph; [holta@uoguelph.ca](mailto:holta@uoguelph.ca), or contact: Dr. Hannah Tait Neufeld, principal investigator, Department of Family Relations and Applied Nutrition, University of Guelph; [hannahtn@uoguelph.ca](mailto:hannahtn@uoguelph.ca).

If you have read the above information and consent to participate, please click “I agree” below to begin the survey, or click “I do not agree” to exit. You may print out this page for future reference if you wish, by clicking “print” below.

🞎 I agree

🞎 I do not agree

## Section 2: Survey Questions

1. Please indicate if this is your first pregnancy or if you have previously been pregnant

This is my first pregnancy

I have been pregnant before

2. How far along are you in your pregnancy?

0-1 month

> 1 month – 2 months

> 2 months – 3 months

> 3 months – 4 months

> 4 months – 5 months

> 5 months – 6 months

> 6 months – 7 months

> 7 months – 8 months

> 8 months – 9 months

Over 9 months (past due date)

3. Where have you looked for nutrition information relating to pregnancy? (Check all that apply)

The internet

Books

Magazines

Family doctor

Midwife

Family

Friends

Brochures

Nine-month calendars

Other (Please specify): ____________________________

I have not looked for nutrition information relating to pregnancy

4. Where did you look for pregnancy-related nutrition information most often? (Only check one box)

The internet

Books

Magazines

Family doctor

Midwife

Family

Friends

Brochures

Nine-month calendars

Other (Please specify): ____________________________

I have not looked for nutrition information relating to pregnancy

5. When have you looked for nutrition information relating to pregnancy? (check all that apply)

Before becoming pregnant

During the first trimester of my pregnancy (0-3 months)

During the second trimester of my pregnancy (3-6 months)

During the 3^rd^ trimester of my pregnancy (6-9 months)

I have not yet looked for nutrition information relating to pregnancy

6. When have you looked for nutrition information relating to pregnancy most often?

Before becoming pregnant

During the first trimester of my pregnancy (1-13 weeks pregnant)

During the second trimester of my pregnancy (14-26 weeks pregnant)

During the 3^rd^ trimester of my pregnancy (27 or more weeks pregnant)

I have not yet looked for nutrition information relating to pregnancy

## Section 3: Demographic Information

1. How old are you?

18-25

26-35

36-45

46-55

56+

2. Which of the following best describes your ethnic background? (Check all that apply)

First Nations/Métis/Inuit

European

African

Southeast Asian

South Asian

Latin American

Middle Eastern

Other (please specify) __________________________________

3. Which of the following best describes your marital status?

Never legally married

Legally married (and not separated)

Separated, but still legally married

Divorced

Widowed

Common-law married

4. Which of the following best describes your HIGHEST level of education?

Some high school

Completed high school

Some college/university

Apprenticeship training and trades

Completed college/university

Some graduate education

Completed graduate education

Professional degrees

5. What is your annual household income?

< $20,000/year

$20,000 - $39,000/year

$40,000 - $59,000/year

$60,000 - $79,000/year

$80,000 - $100,000/year

$100,000 - $149,000/year

$150,000 - $199,000/year

$200,000/year or more

## Section 4: Recruitment for Interviews

The researcher would like to conduct telephone interviews with 12-15 women who are pregnant for the first time to learn more about their experiences with using the internet to find pregnancy-related nutrition information. If you are interested in participating or learning more about this opportunity, please leave your contact information below.

Note: This contact information will be kept separate from these survey answers to protect your identity.

Email: _______________________________

## Section 5: Thank you & Debriefing

Thank you for taking the time to complete this survey. The responses from this survey will be compiled to determine which sources pregnant women are using most often for nutrition information, and when they tend to look for this information. Your responses will help to guide the next part of the research project, which will explore women’s experiences using the internet to find pregnancy-related nutrition information. You have made a valuable contribution to research on prenatal nutrition.

If you would like to receive a summary of the research findings from this survey, please check the box below and leave your email address

🞎 I would like to receive the results from this survey

Email: ________________________

The following is a list of credible online sources of nutrition information for pregnant women:

- Health Canada: <http://www.hc-sc.gc.ca/fn-an/nutrition/prenatal/index-eng.php>
- Dietitians of Canada: <http://www.dietitians.ca/Your-Health/Nutrition-A-Z/Pregnancy.aspx>
- Public Health Agency of Canada: <http://www.phac-aspc.gc.ca/hp-gs/already-deja-eng.php> & <http://www.phac-aspc.gc.ca/hp-gs/guide/01_pn-np-eng.php>
- What to Expect: <http://www.whattoexpect.com/pregnancy/eating-well/pregnancy-diet.aspx>

Thanks again ☺
